# Hybrid Density Functional Investigation of Cu Doping Impact on the Electronic Structures and Optical Characteristics of TiO_2_ for Improved Visible Light Absorption

**DOI:** 10.3390/ma15165645

**Published:** 2022-08-17

**Authors:** Mohammed Benali Kanoun, Adil Alshoaibi, Souraya Goumri-Said

**Affiliations:** 1 Department of Physics, College of Science, King Faisal University, P.O. Box 400, Al-Ahsa 31982, Saudi Arabia; 2 Physics Department, College of Science, Alfaisal University, P.O. Box 50927, Riyadh 11533, Saudi Arabia

**Keywords:** electronic structure, optical absorption, visible light responsive photocatalyst, Cu doping, hybrid functional density functional theory

## Abstract

We report a theoretical investigation of the influence of Cu doping into TiO_2_ with various concentrations on crystal structure, stability, electronic structures and optical absorption coefficient using density functional theory via the hybrid formalism based on Heyd Scuseria Ernzerhof. Our findings show that oxygen-rich environments are better for fabricating Cu-doped materials and that the energy of formation for Cu doping at the Ti site is lower than for Cu doping at the O site under these environments. It is found that Cu doping introduces intermediate bands into TiO_2_, narrowing the band gap. Optical absorption curves show that the Cu-doped TiO_2_ can successfully harvest visible light. The presence of widely intermediate bands above the valence-band edge could explain the increase in the visible light absorption range. However, the intensity of visible light absorption rises with the increase in doping concentration.

## 1. Introduction

Clean and renewable energy technology has sparked a lot of consideration in recent years as a way to create a more sustainable economy and reduce pollution [1,2,3]. Clean hydrogen (H_2_) is seen as a promising alternative energy source for the future because of its high energy density and lack of pollution [3]. Photovoltaic electrolysis, photoelectrochemical cells, and photocatalysts have all been used to investigate a variety of water-splitting systems [4]. Photocatalytic water splitting with unlimited solar energy has attracted much interest since it is an encouraging technique for producing hydrogen. To accomplish the 10% solar energy to hydrogen conversion efficiency objective for useful applications, a significant number of photocatalyst materials have been created over the last few decades [5,6]. Metal oxide photocatalysts are of interest because they are inexpensive, widespread on the earth, and, in many cases, non-toxic. Titanium dioxide (TiO_2_) is considered among the most exploited potential photocatalyst compound for water splitting, due to its excellent photocatalytic activity, and large-term stability opposite to photo and chemical degradation [7]. Nevertheless, because of a wide band gap (~3.20 eV) of pristine TiO_2_, ultraviolet (UV) light is the only type of light that can be absorbed, accounting for only 5% of solar energy [4]. 

Many alternative ways were explored to increase the visible light range for photocatalytic performance [8]. Doping a large band gap oxide semiconductor as a host compound with metals [8,9,10,11] or nonmetals [12,13] is among the most effective way to lower the band gap and boost photocatalytic activity in visible light by altering the band structure. The substitution of element dopants into the TiO_2_ structure results in the generation of new in-gap states, implying the increased performance of photocatalytic and absorption of visible light. Furthermore, owing to the development of carrier trapping sites by element dopants, the electron–hole recombination rate can be efficiently lowered [14]. The copper (Cu) element is a less expensive and more accessible alternative to elements such as Ag and Au, as well as Pt group noble metals [15,16,17,18,19]. It was revealed that, when Cu is doping into TiO_2_, Cu_2_O clusters are created on the surface that can participate in photocatalytic activity [16,17]. Moreover, Cu doping into TiO_2_ was examined for a wide range of photocatalytic areas of application, including production of hydrogen [20,21,22,23], conversion of CO_2_ [24], dye deterioration, and organic pollutant degradation. However, various experimental [16,17,18,19,20,21,22,23,24,25] and theoretical [25,26,27,28,29,30,31] studies on Cu doping have recently been published, suggesting that it exhibits increased photocatalytic activity of TiO_2_ in the visible area. Recently, Khlyustova et al. [32] synthesized the doping of TiO_2_ with various metal dopants using the sol–gel technique. The introduction of Cu causes a deformation of the TiO_2_ crystal structure, modifying its surface properties, and lowering the band gap, resulting in a 70% increase in photocatalytic activity [32]. More recently, Bhattacharyya et al. [24] have reported that under visible light irradiation, a Cu-doped TiO_2_ demonstrates high efficiency and selective photo-conversion of carbon dioxide to methane. They also examined the parameters that govern the photocatalysis of Cu-doped TiO_2_ systems, such as structure–activity correlation factors. Based on the experimental results, greater emphasis should be directed to Cu doping into TiO_2_ for different Cu compositions to produce photocatalyst materials with the response to visible light and strong activity. In this work, we aim to study the effect of substituting Ti with Cu atoms at various contents on the structural stability, electronic properties and visible light absorption of TiO_2_. Therefore, we applied state-of-the-art first-principle computations in the context of the hybrid functional density functional theory method.

## 2. Materials and Methods

The Quantum Atomistix ToolKit (QuantumATK) software [33] based on local combination of the atomic orbitals method was used to explore first-principles calculations using the generalized gradient approximation (GGA) of the Perdew, Burke, Ernzerhof (PBE) exchange-correlation potential in the density functional theory (DFT) framework [34]. For defining the ion nuclei and valence electrons interaction, the norm-conserving PseudoDojo [35] pseudopotential was adopted. Energy convergence was calculated using self-consistent field computations with a tolerance limit of 10^−8^ Ha. The basis set was set to PseudoDojo-medium with a mesh cut-off energy of 90 Ha. The stability arrangement of atoms and lattice parameters were obtained by lowering the total energy of the system using the Broyden–Fletcher–Goldfarb–Shanno (LBFGS) method until the force on each atom was reduced below 0.05 eV/Å. A 4 × 4 × 3 Monkhorst-Pack [36] k-grid is utilized for geometry optimization, while a 10 × 10 × 8 grid is employed for electronic property computations. The more accurate hybrid density functional as described by Heyd–Scuseria–Ernzerhof (HSE06) was applied to predict the appropriate band gap energy and optical characteristics [37,38] of semiconductors. The exchange-correlation energy in the HSE function was divided into short- (sr) and long-range (lr) parts, which is defined as

(1) $$E_{xc}^{HSE}\left(\mu ,\alpha \right)=\alpha E_{x}^{HF,sr,\mu }+\left(1-\alpha \right)E_{x}^{PBE,sr,\mu }+E_{x}^{PBE,lr,\mu }+E_{c}^{PBE}$$

where μ is the parameter that defines the range separation of Coulomb kernel (μ = 0.2/Å) and α is the mixing parameter of 0.20.

The electronic band structure of systems is connected with the dielectric function ε(ω) = ε_1_ (ω)+iε_2_ (ω), which is estimated using the linear response method. We used the intra- and inter-band transitions in the dielectric function to calculate the optical response of investigated systems. The phonon contribution is ignored in the current approach. However, the direct transition zone between the occupied state in the valence band and the unoccupied state in the conduction band was considered. The following equation can be used to calculate the imaginary component of the dielectric function ε_2_ (ω) [39,40]:

(2) $$\varepsilon_{2}^{ij}=\frac{4\pi^{2}e^{2}}{Vm^{2}\omega^{2}}\times \underset{nn^{\prime }\sigma }{\sum }\langle kn\sigma |P_{i}|kn^{\prime }\sigma \rangle \langle kn^{\prime }\sigma |P_{j}|kn\sigma \rangle \times f_{kn}\left(1-f_{kn^{\prime }}\right)\delta \left(E_{kn^{\prime }}-E_{kn}-\hbar \omega \right)$$

where e and m denote the electron’s charge and mass, respectively. ω is the frequency of incident photons on the crystal. The volume of the unit cell is denoted by V. |knσ〉 denotes the crystal’s wave function and k denotes its momentum with σ as spin. The $f_{\mathrm{kn}}$ Fermi distribution function allows for the calculation of transitions between occupied and unoccupied states. The optical response is activated by the electric dipole transitions present in the middle of VB and CB. 

Using the Kramers–Kronig relation, the real part $\varepsilon_{1}\left(\omega \right)$ of the dielectric function is calculated from the imaginary part [39,40]:

(3) $$\varepsilon_{1}\left(\omega \right)=1+\frac{2}{\pi }P\int_{0}^{\infty }\frac{{\omega \prime \varepsilon }_{2}\left(\omega \prime \right)}{\omega^{\prime 2}-\omega^{2}}d\omega \prime$$

where ‘P’ represents the principal value of integral.

## 3. Results and Discussion

**Figure 1:**
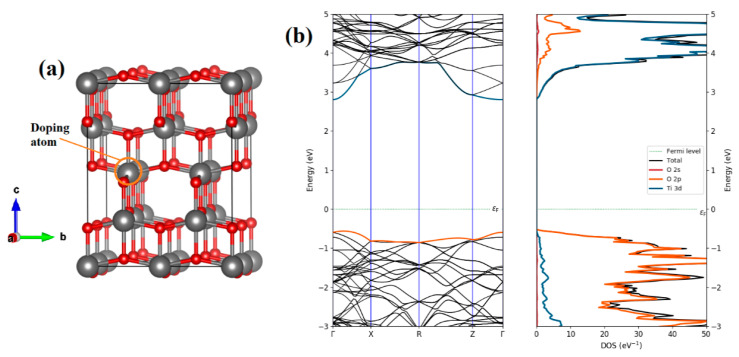
(**a**) The optimized geometric structure of 2 × 2 × 1 supercell and (**b**) computed electronic band structure and total/partial of pure TiO_2_. (Ti in dark gray, O in red, orange circle of doping atom).

For the pristine anatase TiO_2_ with the space group I41/amd (see Figure 1a), the computed lattice constants are found to be a = b = 3.781 Å, c = 9.499 Å, which agrees with the experimentally reported values (a = b = 3.7842 Å, c = 9.5146 Å) [41]. As depicted in Figure 1b, the band structure of pure TiO_2_ exhibits that the valence band maximum (VBM) is situated simultaneously at the Γ- and X-points, while the conduction band minimum (CBM) is located at the Γ-point displaying an indirect semiconductor behavior. The predicted band gap value of 3.37 eV is more consistent with the experimental data (3.2 eV) [25] and earlier studies [25,26,27,28,42,43]. The DOS curves of undoped anatase reveal that the 2p states of oxygen dominate the edge of the valence band whilst the empty 3d states of titanium dominate the conduction band’s minimum. A periodic 2 × 2 × 1 supercell with 48 atoms was used for Cu-doped TiO_2_, as illustrated in Figure 1a. To define the preferable sites for Cu dopants, the formation energies are computed for two configurations considering, one substitutional Cu at the Ti site (labeled by CuTi), and one substitutional Cu at the O site (labeled by CuO). The formation energy of the doped configurations is estimated by [44,45]

(4) $$E_{f}=E_{\mathrm{doped}}-E_{\mathrm{undoped}}-n_{\mathrm{Cu}}\mu_{\mathrm{Cu}}+n_{\mathrm{Ti}}\mu_{\mathrm{Ti}}+n_{O}\mu_{O}$$

where E_undoped_ and E_doped_ represent the respective total energies of undoped and doped TiO_2_ supercell. n denotes the number of added or removed dopant and host ions. The μ_Cu_, μ_Ti_ and μ_O_ represent the chemical potential of the Cu, Ti and O atoms, respectively, which depend on various experimental conditions. For a Ti-rich and an O-rich environment, the μ_Ti_ and μ_O_ can be taken in the ground state with the energy of bulk Ti and O_2_ molecules, respectively. The μ_Ti_ under O-rich conditions and μ_O_ under Ti-rich environment can be estimated by the growth condition:

(5) $$\mu_{\mathrm{Ti}}+2\mu_{O}\approx \mu_{{\mathrm{TiO}}_{2}}$$

Note that $\mu_{{\mathrm{TiO}}_{2}}$ is determined by the total energy per one formula unit of stoichiometric TiO_2_.

The $\mu_{\mathrm{Cu}}$ can be computed using the formula below:

(6) $$2\mu_{\mathrm{Cu}}+3\mu_{O}\approx \mu_{{\mathrm{Cu}}_{2}O_{3}}$$

where $\mu_{{\mathrm{Cu}}_{2}O_{3}}$ is calculated from the energy of the Cu_2_O_3_.

The computed energies of formation for single Cu doping into TiO_2_ at both the Ti and O sites are gathered in Table 1. It indicates that the Cu dopant preferred to replace the Ti site both under Ti-rich and O-rich environments which is the most energetically favorable structure.

**Figure 2:**
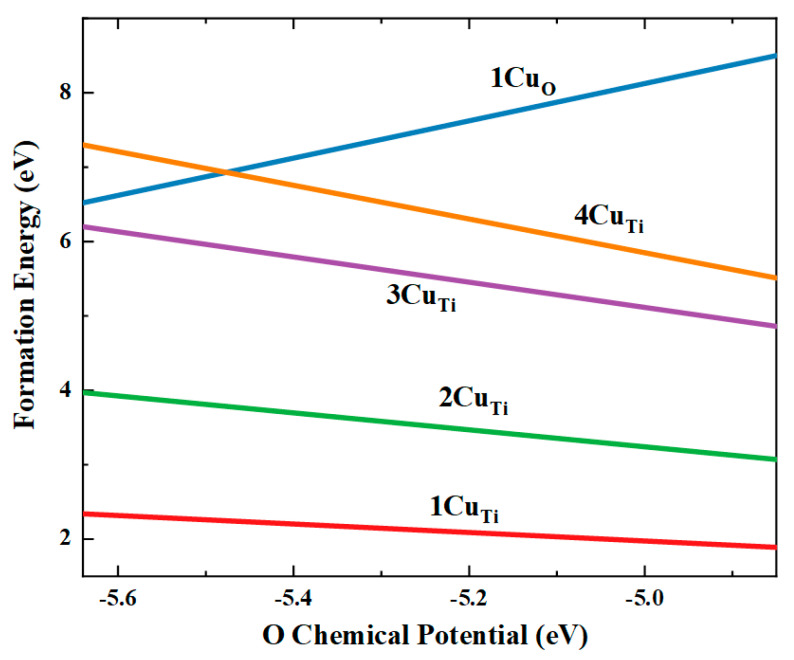
Predicted energies of formation versus the oxygen chemical potential for Cu doping configurations.

It demonstrates that under both the Ti-rich and O-rich environments, the Cu dopant prefers to substitute the Ti site, which is the most thermodynamically favorable structure. It is observed that the configuration CuTi has lower formation energy in O-rich circumstances, and Cu can barely dope in any other TiO_2_ locations in an O-rich environment. We found that Cu impurity can also replace the O atom under Ti-rich circumstances when Cu and Ti are both adequate. The substitution of O by Cu into the TiO_2_ lattice causes the surrounding Ti atoms to migrate outward significantly due to the substantially higher ionic radius than O. Therefore, we only concentrated our investigation on the most stable CuTi. Moreover, the energies of formation for higher dopant concentrations are estimated by replacing two substitutional Cu at the Ti sites (labeled by 2CuTi), three substitutional Cu at the Ti sites (labeled by 3CuTi), and four substitutional Cu at the Ti sites (labeled by 4CuTi). It can also be seen from Table 1 that the formation energy of distinct Cu doping configurations is lower than of the CuO configuration under O-rich conditions, meaning that all Cu doping systems can be obtained much easier. Figure 2 shows the change in the energy of formation of Cu doped TiO_2_ with different concentrations versus O chemical potential. According to an analysis of these findings, it is energetically preferable for all Cu doping into TiO_2_ at Ti site configurations to form under O-rich growth conditions rather than under a Ti-rich environment. It is observed that the formation energy for the TiO_2_ doping with one Cu and two Cu at the Ti sites is low in the O-rich environment than that of the three Cu and four Cu, suggesting that 1CuTi is a relatively stable dopant and easily spontaneously forms under thermodynamic equilibrium conditions.

After the relaxation of geometries, the predicted lattice parameters of the investigated configurations are gathered in Table 1. Our results show that the lattice parameter a is slightly larger whereas c is slightly less than that of pure TiO_2_. The calculated length between Cu and the neighboring O atom is 1.874 Å, which is slightly smaller than the origin Ti–O bond of 1.986 Å owing to the ionic radii differences. Bader charge assessments indicate the discrepancy between the Cu dopants and the Ti and O atoms in the supercells for various configurations: the Ti and O elements in the pure system have charges of +2.21, −1.106e, respectively, whereas the Cu atoms doping into TiO_2_ supercells have values of +1.35e.

**Figure 3:**
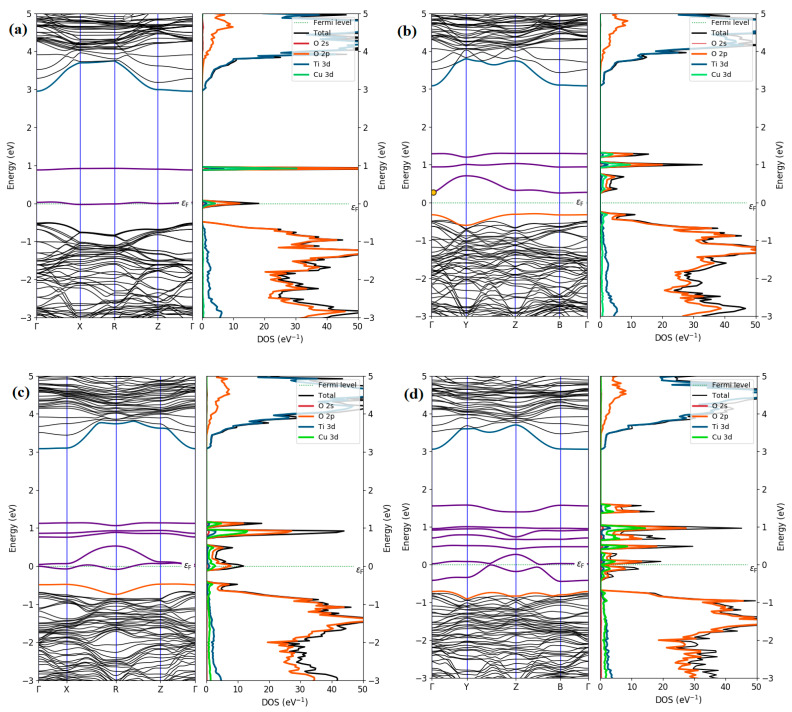
Calculated band structure and density of states of (**a**) 1Cu_Ti_, (**b**) 2Cu_Ti_, (**c**) 3Cu_Ti_, and (**d**) 4Cu_Ti_, doped TiO_2_.

To explore the effect of Cu impurities on the change of the electronic properties, we computed the electronic band structures and total and partial density of states (DOS) of Cu doping configurations, as illustrated in Figure 3. When incorporating a Cu defect in the TiO_2_ structure, two new defect states appear in the band gap region above CBM, as displayed in Figure 3a, which evidences that doping with a Cu atom will make TiO_2_ act as a p-type semiconductor. This is due to the fact that doping with Cu releases one more valence electron than the host Ti. Cu doping may be able to bring the absorption peak of TiO_2_ closer to the visible range by introducing defect states into the band gap. This behavior was also obtained in previous theoretical results [25,26,27,28,29,30,31].

By doping more Cu atoms in TiO_2_ lattice structures, the electronic structures show similar characteristics to that of single Cu doping, except that more impurity states appear above the VBM due to variation in the concentration of Cu dopants. The DOS and PDOS of single Cu doping display that two distinct in-gap peaks are formed between VBM and CBM, in which the first peak is partially occupied around the Fermi and the second peak is unoccupied. Moreover, the composition of the VBM is still dominated by O 2p states with small contributions of Cu 3d and Ti 3d states, but the CBM does not differ from that of pristine TiO_2_. The hybridization states produced between the Cu 3d and O 2p levels constitute the impurity state. As the amount of doping is increased, we observe that the acceptor state is formed containing a large contribution of 2p states and 3d states of O and Cu, respectively, together with a small component of Ti 3d states, indicating that the O 2p states hybridize with Cu 3d states.

**Figure 4:**
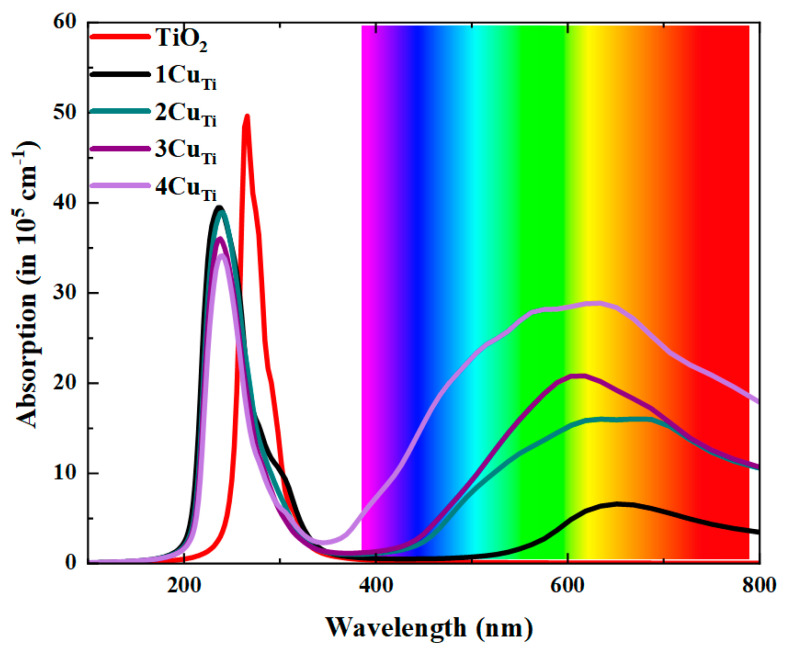
Computed optical absorption coefficients of undoped and Cu-doped TiO_2_ with different configurations at the HSE06 level.

To examine the absorption characteristics in undoped and Cu-doped TiO_2_ configurations, we determined optical absorption coefficient spectra as a function of wavelength using the HSE06 hybrid function, as illustrated in Figure 4. The analysis of optical absorption features shows that the pure TiO_2_ can only collect in the UV region and has no visible light absorption response. The single Cu doping generates a little red shift of the absorption peak because of the defect intermediate bands. From the density of states, it is observed that the impurity states are induced by Cu doping, showing little visible light absorption. With the increase in Cu substitution concentrations (2CuTi, 3CuTi, 4CuTi), the excitation energy from the impurity state in band gag to CBM is commensurate with the augmentation of visible light absorption in the region. Importantly, the optical curves of 4CuTi between 400 and 800 nm are found to be much higher than that of 1CuTi. The main difference here can be attributed to the increase in defect energy states around and above Fermi in the increase in Cu concentrations as compared to pristine.

**Figure 5:**
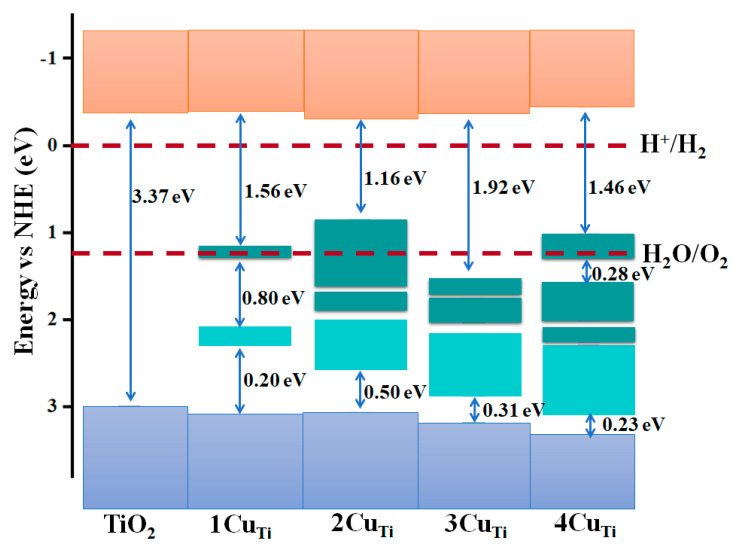
The band edge position of the pristine and Cu-doped TiO_2_ configurations with regards to reduction and oxidation potentials of water. The CBMs and VBMs are designated with orange and light blue regions, respectively, while the intermediate energy bands are identified with broad light and dark green sections.

To describe the influence of Cu doping into TiO_2_ on the photocatalytic characteristics, we calculated the relative locations of band edges of pristine TiO_2_ in relation to the reduction potential of water using the expression [46]:

(7) $$E_{\mathrm{CBM}}=\chi -0.5E_{g}+E_{0}$$

(8) $$E_{\mathrm{VBM}}=E_{\mathrm{CBM}}+E_{g}$$

where $E_{g}$ is the band gap obtained at the HSE06 level of approximation, χ represents the tin dioxide catalyst’s absolute electronegativity, and E_0 denotes the free electron energy on the hydrogen dimension (~4.5 eV). The energy of VBM (E_VBM_) was calculated from the respective band gap and energy of CBM (E_CBM_). According to calculations, the CBM of TiO_2_ is found to be −0.385 eV, and the VBM is 2.99 eV. Our results agree well with the reported results [47]. Figure 5 represents the alignment of the band edge of undoped and doped TiO_2_ in comparison to the redox and oxidation potentials of the water. Notice that the band edges of the water reduction and oxidation levels are evaluated in relation to the normal hydrogen electrode (NHE) potentials: the decrease level (H+/H_2_) is situated at 0 eV, while the oxidation level (H_2_O/O_2_) is located at 1.23 eV [48]. In the usual representation, the CBM is “above” the reduction of the water (H+/H2) level, whereas the VBM is “below” the oxidation of the water (H_2_O/O_2_). 

This means that while pristine TiO_2_ seems to have a high reduction/oxidation capacity, it is unable to capture solar light successfully. It is observed that Cu doping leads to induce intermediate bands, which could enable transitions of charge carriers from the valence region to the unfilled intermediate bands or from the unfilled intermediate bands to the conduction region, as well as transitions within the partially filled intermediate band. These intermediate bands contribute to narrowing the band gap to 1.56 eV, 1.16 eV, 1.92 eV and 1.46 for 1CuTi, 2CuTi, 3CuTi, and 4CuTi, respectively.

## 4. Conclusions

Hybrid density functional computations were performed to study the incorporation of Cu with different concentrations at Ti sites of anatase TiO_2_ on the crystal lattice structure, using DFT to explain how they affect the structure stability, electronic, and optical properties. We found that under O-rich and Ti-poor circumstances, it was more energetically favorable to generate Cu doping into TiO_2_ at the Ti site than at the O site. It is observed that Cu doping tuned the band gap, which directly affects the electronic and optical absorption. Therefore, it can thereby lower the band gap energy of TiO_2_ by introducing intermediate bands. Optical absorption curves reveal that the Cu-doped TiO_2_ can successfully harvest visible light, resulting in a rise in visible light absorption. The existence of widely intermediate bands above the edge of the valence band could explain the increased visible light absorption. However, the absorption of visible light is improved by increasing the doping concentration.

## Figures and Tables

**Table 1:** The optimized lattice parameters, cell volume and the formation of defects for pristine and modified TiO_2_.

	a (Å)	c (Å)	V (Å^3^)	E_f_ (eV)	
				Ti-rich	O-rich
1CuTi	3.785	9.456	541.95	2.34	1.89
1CuO	3.812	9.430	548.17	6.52	8.50
2CuTi	3.782	9.471	541.86	3.97	3.07
3CuTi	3.782	9.428	540.05	6.20	4.86
4CuTi	3.761	9.430	539.23	7.30	5.51
